# Reducing culture medium nitrogen supply coupled with replenishing carbon nutrient simultaneously enhances the biomass and lipid production of *Chlamydomonas reinhardtii*

**DOI:** 10.3389/fmicb.2022.1019806

**Published:** 2022-09-26

**Authors:** Shiyan Zheng, Shangyun Zou, Hongyan Wang, Tian Feng, Shourui Sun, Hui Chen, Qiang Wang

**Affiliations:** ^1^Jiangsu Key Laboratory of Marine Bioresources and Environment, Jiangsu Key Laboratory of Marine Biotechnology, Jiangsu Ocean University, Lianyungang, China; ^2^State Key Laboratory of Crop Stress Adaptation and Improvement, School of Life Sciences, Henan University, Kaifeng, China; ^3^Academy for Advanced Interdisciplinary Studies, Henan University, Kaifeng, China

**Keywords:** *Chlamydomonas reinhardtii*, carbon and nitrogen levels, lipid production, microalgal biomass, fatty acid compositions

## Abstract

*Chlamydomonas reinhardtii* is a model strain to explore algal lipid metabolism mechanism, and exhibits great potentials in large-scale production of lipids. Completely lacking nitrogen is an efficient strategy to trigger the lipid synthesis in microalgal cells, while it always leads to the obvious reduction in the biomass. To illustrate the optimal culture substrate carbon (C) and nitrogen (N) levels to simultaneously stimulate the growth and lipid production of *C. reinhardtii*, cells were cultivated under altered C and N concentrations. Results showed that replenishing 6 g/L sodium acetate (NaAc) could increase 1.50 and 1.53 times biomass and lipid productivity compared with 0 g/L NaAc treatment (the control), but total lipid content slightly decreased. Reducing 75% of basic medium (TAP) N level (0 g/L NaAc + 0.09 g/L NH_4_Cl treatment) could promote 21.57% total lipid content in comparison with the control (containing 0.38 g/L NH_4_Cl), but decrease 44.45% biomass and 34.15% lipid productivity. The result of the central composite design (CCD) experiment suggested the optimum total lipid content together with higher biomass and lipid productivity could be obtained under the condition of 4.12 g/L NaAc and 0.20 g/L NH_4_Cl. They reached 32.14%, 1.68 g/L and 108.21 mg/L/d, and increased by 36.77%, 93.10% and 1.75 times compared with the control, respectively. It suggests moderately increasing C supply and decreasing N levels could synchronously improve the biomass and lipid content of *C. reinhardtii*.

## Introduction

As one of the dominant storage forms of carbon and energy in algal cells, lipids are an essential nutrient in the human diet, an important source of chemical raw materials, and a promising feedstock of renewable energy, which exhibit considerable roles in many areas such as food, energy, environment, etc. (Sajjadi et al., 2018; Munoz et al., 2021; Udayan et al., 2022). With the rapid increase in world population, urbanization and industrialization, the traditional lipid resources have failed to meet the massive market demands (Ma et al., 2022; Udayan et al., 2022). Most microalgae could effectively biosynthesize substantial amounts of lipids and other high-value active substances with a short life cycle, high photo-conversion efficiency, strong adaptability, etc., which have been reputed to be a new generation of lipid bio-resources (Sajjadi et al., 2018; Chen and Wang, 2022).

Considering microalgal lipids are much safer than animal oils and traditional chemical feedstock, and cleaner than fossil fuels, it has attracted worldwide concerns (Shi et al., 2017; Li et al., 2021). More importantly, microalgae display high plasticity, and lipid levels are closely associated with growth conditions. The lipid production can be further obviously enhanced under specified conditions (Hu et al., 2017; Levasseur et al., 2020). It was confirmed that diverse stress conditions facilitate the lipid synthesis of microalgae, which include nutrient depletion, increasing culture temperature and light intensity, high salinity, and so on (Brindhadevi et al., 2021; Chen and Wang, 2021). Nitrogen deficiency is supposed to be the most efficient strategy for stimulating the lipid biosynthesis of microalgae, whereas it is at the expense of reducing biomass, obtaining lower lipid productivity (Chen and Wang, 2021). Other studies found that a two-stage nitrogen depletion method could achieve the enhancements of lipids and biomass (Gao et al., 2019; Aziz et al., 2020), but it would remarkably increase production costs resulting from expending more water, nutrient ingredients and the power for collecting the first stage algal cells. Hence, to satisfy the large market requirements of algal lipids, it is still an urgent problem that how to efficiently improve microalgal lipid content accompanied by high biomass production.

The microalgal strain of *Chlamydomonas reinhardtii* can generate around 20% total lipids and grows fast with a short growth period under normal conditions, which has been comprehensively deemed to be a model organism to research the mechanisms of lipid metabolism in microalgae (Moellering et al., 2009). C*hlamydomonas* cells are also extensively employed as a bio-factory to synthesize various high bioactive components, and exhibit increasingly promising potentials in health foods, biofuels, pharmaceuticals, and nutraceuticals. It is well-known that the nutrients of both carbon (C) and nitrogen (N) display considerable impacts on stimulating the biomass and lipid accumulations of many algal strains (Sajjadi et al., 2018; Yaakob et al., 2021; Ma et al., 2022). To date, for *C. reinhardtii*, the most related literature primarily focused on the influences of nitrogen stress or coupled with carbon sources supplementation on its lipid production, and the nitrogen starvation was usually induced by a complete lack of nitrogen sources (Goodson et al., 2011; Ramanan et al., 2013; Smith and Gilmour, 2018; Chen and Wang, 2021). It needs further work to elucidate the optimal C and N levels for concurrently improving the biomass and lipids of *C. reinhardtii*.

Keeping a small amount of nitrogen nutrients in culture substrate might be more conducive to the enhancement of lipid yield. Li et al. (2019) pointed out that the proportion of carbon to nitrogen greatly affects the nutrient assimilation of microalgae. Sajjadi et al. (2018) found that the combination of optimized carbon source levels and low-nutrition conditions might significantly enhance the production efficiency of microalgae and shorten the life cycle. Li et al. (2020) reported that the marked improvements in biomass, carbohydrates and polyunsaturated fatty acids were observed in *Porphyridium purpureum* by replenishing appropriate glycerol under lower nitrogen content conditions.

In addition, the optimum C and N concentrations of diverse microalgae are species/strain-specific (Perez-Garcia et al., 2011; Ma et al., 2022). To obtain the desired biomass and lipid yield, the growth performance, lipid property and fatty acid profiles of *C. reinhardtii* were investigated under varied medium C and N levels. This study aims to explore the potential of *C. reinhardtii* in lipid production from an optimizing substrate C-N nutrients perspective.

## Materials and methods

### Algal strain and cultivation conditions

The algal strain of *Chlamydomonas reinhardtii* (CC-125), provided by Institute of Hydrobiology, Chinese Academy of Sciences, was used in this research. Cultures were incubated in Tris-acetic acid- phosphate (TAP) medium, the constituents of which in 1 L contained 2.42 g tris powder, 0.38 g NH_4_Cl, 0.10 g MgSO_4_⋅7H_2_O, 57.00 mg CaCl_2_⋅2H_2_O, 0.11 g K_2_HPO_4_, 54.00 mg KH_2_PO_4_, 50.00 mg EDTANa_2_, 22.00 mg ZnSO_4_⋅7H_2_O, 11.40 mg H_3_BO_3_, 5.06 mg MnCl_2_⋅4H_2_O, 1.61 mg CoCl_2_⋅6H_2_O, 1.57 mg CuSO_4_⋅5H_2_O, 1.10 mg (NH_4_)_2_Mo_7_O_24_⋅4H_2_O, 4.99 mg FeSO_4_⋅7H_2_O and 1 mL glacial acetic acid (Gorman and Levine, 1965). The working volume was 200 mL in 250 mL Erlenmeyer flask, and the initial inoculum was 5% (v/v) of logarithmic phase algal cells (∼ 0.03 g/L). *C. reinhardtii* was cultivated in a shaker at 25°C with 50 μE/m^2^/s light intensity and 14/10 h light/dark cycle.

### Experimental design

To clarify the effects of carbon (C) and nitrogen (N) levels of culture medium on the growth and lipid characteristics of *C. reinhardtii*, the single factor experiment, two-factor experiment and response surface method (RSM) were conducted in this research, and the cultures cultivated in TAP medium were considered as the control (containing 0 g/L NaAc and 0.38 g/L NH_4_Cl). Firstly, to explore the impacts of altered carbon levels of culture substrate on the growth and lipid production of *C. reinhardtii*, sodium acetate (NaAc) was used in this study since the carbon source in TAP medium is acetic acid. Algal cells were cultivated in six various NaAc concentrations, including 0, 2, 4, 6, 8 and 10 g/L (w/v). Secondly, to further probe the influences of reducing nitrogen level coupled with supplementing carbon source on the growth performance and lipid property of *C. reinhardtii*, the two-factor experiment was carried out, where carbon levels contained 0, 2, 4 and 6 g/L of NaAc, and nitrogen concentrations included 0.09, 0.19 and 0.28 g/L (w/v) of NH_4_Cl. The content of NH_4_Cl in TAP medium was 0.38 g/L. This experiment contained 12 treatments in total, which were orderly named G1-G12 as described in Table 1.

**TABLE 1:** The design of two-factor experiment.

NH_4_Cl concentration (g/L)	NaAc concentration (g/L)			
	
	0	2	4	6
0.09	G1	G4	G7	G10
0.19	G2	G5	G8	G11
0.28	G3	G6	G9	G12

Finally, to reveal the optimal carbon and nitrogen levels of culture medium, the central composite design (CCD) of RSM was employed for optimization experiment, the design and statistical analysis of which were conducted by Design-Expert 13 software (Stat-Ease Inc., America). Each factor was set to five levels, including plus and minus alpha (axial points), plus and minus 1 (factorial points) and the center point. The factor ranges were fixed by entering ± 1 levels, the carbon of which were 2 g/L (−1) and 6 g/L (+ 1) NaAc, and 0.09 g/L (−1) and 0.28 g/L (+ 1) NH_4_Cl for nitrogen, respectively. As the replicates of the center points were designed five times, a total of thirteen experimental runs were performed with three different responses of biomass, total lipid content and lipid productivity. All experiments were conducted three times.

### Analysis of *Chlamydomonas reinhardtii* growth

To evaluate the growth of *C. reinhardtii* under diverse culture conditions, the biomass and chlorophylls of cultures were determined in this study. The biomass was measured by a gravimetric method and displayed as dry cell weight (DCW). Each sample of 2 mL microalgal cells was centrifuged at 3,000 g for 5 min and collected in pre-weighed centrifuge tube. Then, all samples were placed into an oven at 65°C with constant weight, and the weight was recorded to calculate DCW. Besides, the specific growth rate (μ) was also computed according to the reported equation by Zheng et al. (2021).

The chlorophyll contents of samples were determined with a modified methanol method (Lichtenthaler and Wellburn, 1983). An aliquot of 1 mL cultures was centrifuged at 3,000 g for 5 min, and replenished the same volume of ∼100% methanol after removing the supernatant. Then, the resuspended samples were placed in 4°C refrigerator for 24 h with keeping darkness. The methanol-extract absorbance at 653 and 666 nm was measured with a Multiskan GO Microplate Reader (Thermo, America).

### Measurement of total lipids

The algal cells cultured for 5 days were collected by centrifugation at 4°C, 3,000 g for 5 min, and dried for 48 h in a vacuum freezing drier (Labconco FreeZone Plus, America). Total lipid content in dried cells was determined with a Chloroform-methanol method (Bligh and Dyer, 1959). A sample of 50 mg dried pellets was mixed with 3 mL chloroform - methanol (2:1, v/v) in a 15 mL tube, and then sonicated for 10 min in an ice-water bath. The supernatant was gathered into a 15 mL pre-weight glass tube by centrifuging at 6,800 g for 10 min. The processes of the above sonication and centrifugation steps were repeated twice for completely extracting lipids. All gathered supernatants were dried at 65°C in an oven. The lipid content was exhibited as DCW percentage (% DCW). The extracted lipids were used for further analyzing fatty acid composition.

### Analysis of fatty acid profile

To analyze the changes of fatty acid compositions in *C. reinharditii* under various cultivation conditions, the fatty acid profiles of the cultures were measured using a gas chromatography (GC) method. Fatty acids in extracted total lipids were firstly converted to fatty acid methyl esters (FAME) according to an improved method reported by Liu (1994). An aliquot of 1 mL 1.0% NaOH-CH_3_OH was added into the 15 mL lipids-contained glass centrifuge tubes while filling nitrogen (N_2_) for 1 min. The mixed solutions were saponified in a water-bath at 75°C for 15 min after sonicating for 5 min in an ultrasonic cleaner. Then, 2 mL HCl-CH_3_OH (5.0%, w/v) was supplemented when the above samples were cooled down to room temperature. The mixtures were aerated with N_2_ and placed into 75°C water-bath for 15 min again. The saponified samples were separated into two phases by adding 1 mL n-hexane after cooling down. The n-hexane layer was gathered in 2 mL GC vials by centrifuging at 1,700 g for 5 min for analyzing fatty acid constituents. The manual procedures of GC were conducted according to the described method by Zheng et al. (2021). The chromatograph peaks were identified based on the retention time of each fatty acid standard (Sigma-Aldrich Supelco 37 component FAME mix, America). The fatty acid content was calculated according to the proportion of each peak area in the total FAME peak areas. All samplings were performed in triplicate.

### Measurements of intracellular soluble sugar and protein contents

An aliquot of 10 mL algal cells after 5 days of cultivation was concentrated by centrifuging at 4°C, 3,000 g for 5 min, and replenished the same volume of ultrapure water to resuspend cells after removing the supernatant. Then, all resuspended samples were sonicated for 10 min in an ice bath after freeze-thawing for three times, and centrifuged at 4°C, 6,800 g for 10 min. The collected supernatants were used for analyzing the contents of intracellular soluble sugar and protein as depicted by Zheng et al. (2022).

### Statistical analysis

Statistical significance was assessed by one-way analysis of variance (ANOVA) using Origin 2021 (OriginLab Corporation, America). The figures were also prepared by Origin 2021.

## Results and discussion

### Effects of various carbon levels on the growth, lipid accumulation and fatty acid compositions of *Chlamydomonas reinhardtii*

#### Growth of *Chlamydomonas reinhardtii* under different carbon levels

**FIGURE 1:**
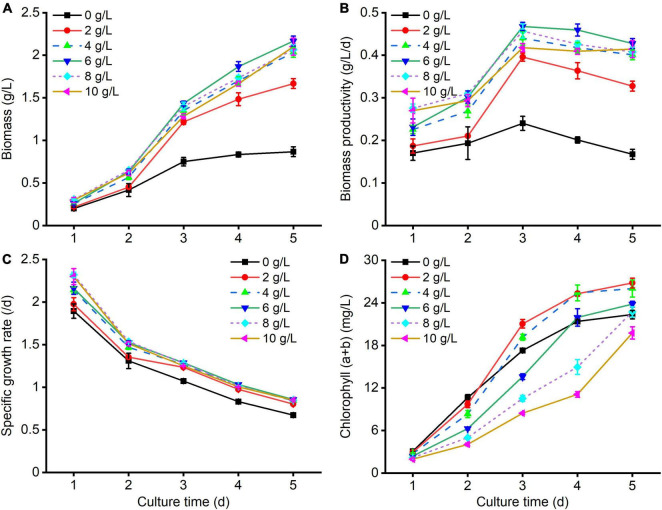
Growth kinetics of *C. reinhardtii* under different carbon levels. **(A–D)** Are the changes of biomass, biomass productivity, specific growth rate, and chlorophyll (*a* + *b*) of *C. reinhardtii* under various NaAc concentrations (0, 2, 4, 6, 8, 10 g/L), respectively. The treatment of 0 g/L NaAc was considered as the control. Error bars indicate standard deviation (*n* = 3). The same as below.

It was observed that *C. reinhardtii* represented diverse growth responses to various carbon levels (Figure 1). As shown in Figure 1A, the biomass of treated algal cells by supplementing NaAc in culture substrate was markedly higher than that of the control (0 g/L NaAc). With the augmentation of NaAc concentration, microalgal biomass and biomass productivity firstly increased and then slightly reduced (Figures 1A,B). After cultivating for 5 days, higher biomass and biomass productivity were obtained under treatment of 6 g/L NaAc, reaching 2.17 g/L and 0.43 g/L/d. They were 1.50 and 1.55 times higher than those of the control, and slightly bigger than the treatments of 4, 8 and 10 g/L NaAc. Besides, the specific growth rates of treated cultures could reach 0.80–0.86/d after incubation of 5 days, which were noticeably higher than that of the control (0.67/d, Figure 1C). This result indicates that there are no significant discrepancies among the biomass of 4–10 g/L NaAc treatments.

Unexpectedly, the chlorophyll (*a* + *b*) levels in treated cells obviously decreased with the increase of NaAc concentration (Figure 1D). After culturing for 5 days, except for 8 g/L and 10 g/L NaAc treatments, the chlorophyll (*a* + *b*) contents in other treatments were higher than that of the control. The maximum chlorophyll (*a* + *b*) content was observed in the treatment of 2 g/L NaAc, slightly higher than 4 g/L NaAc, which was 26.79 mg/L and improved by 19.95 and 12.52% in comparison with the control and 6 g/L NaAc, respectively. Similar result was also found in other microalgal strains (Liu et al., 2008; Yan et al., 2012; Zheng et al., 2017; Cecchin et al., 2018).

Above results illustrate that moderately increasing carbon level of culture medium by adding NaAc could significantly enhance the biomass production of *C. reinhardtii*. It should be ascribed that *C. reinhardtii* efficiently assimilated the carbon source from exogenous NaAc to cell growth (Perez-Garcia et al., 2011). Zheng et al. (2017) reported a similar conclusion that the improved biomass of *Chlorella* sp. was triggered by supplementing NaAc in culture substrate. The decrements of chlorophyll (*a* + *b*) in higher NaAc concentration treatments might be attributed to the photoinhibition caused by excessive organic carbon sources or the altered light condition with the increase of microalgal cell density (Yan et al., 2012; Chapman et al., 2015). Chapman et al. (2015) pointed out that, under the presence of acetate, the reduction in photosynthesis capacity of *C. reinhardtii* was observed when compared with phototrophic cultures, and *C. reinhardtii* could bypass the pathway of CO_2_ fixation and directly absorb acetic acid as the carbon source to stimulate the accumulation of algal biomass.

#### Lipid accumulation and fatty acid compositions of *Chlamydomonas reinhardtii* under different carbon levels

**FIGURE 2:**
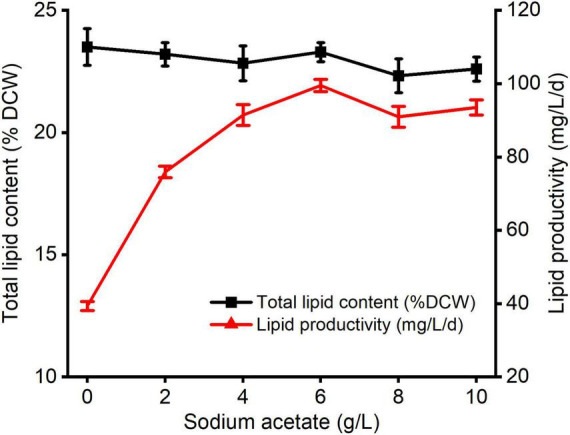
The changes of total lipid content and lipid productivity in *C. reinhardtii* under different carbon levels.

The changes in lipid production of *C. reinhardtii* under varied carbon levels were shown in Figure 2. There were non-significant differences between diverse NaAc treatments and the control. The maximum total lipid value was 23.29% and observed at 6 g/L NaAc treatment, which was 0.90% lower than that of the control. The optimal lipid productivity was also achieved under treatment of 6 g/L NaAc, reaching 99.53 mg/L/d, and improved by 1.53 times compared with the control. This result reveals that supplementing 2–10 g/L NaAc cannot stimulate total lipid accumulation of *C. reinhardtii*, but lipid productivity dramatically improved.

This result indicates that reasonably increasing medium carbon levels with supplementation of NaAc could markedly enhance the lipid productivity of *C. reinhardtii*, but have no obvious positive impacts on triggering lipid synthesis. The slight reductions in total lipids were not in accordance with the results described by previous studies (Ramanan et al., 2013; Zheng et al., 2017; Cecchin et al., 2018). It might be due to the different algal strains, culture conditions or cultivation periods. Zamzam et al. (2022) pointed out that the remarkable improvement of starch level in *C. reinhardtii* was induced by 30 mM NaAc treatment (around 2.46 g/L NaAc), whereas the slight decrement of triacylglyceride content was also observed within incubating for 3.5–4 days. But, it was promoted when the cultivation time increased to 10 days. Based on the growth dynamics under various carbon contents (Figure 1), it was speculated that most absorbed carbon source by *C. reinhardtii* from exogenous NaAc was utilized for cell growth or other active compounds generations such as carbohydrate and protein. The specific mechanism needs more work to be done.

The responses of fatty acid components of *C. reinhardtii* to varied carbon levels were shown in Table 2. The dominant fatty acids of *C. reinhardtii* contained C16:0, C18:1, C18:2 and C18:3, the ratios of which all exceeded 5% in total fatty acids. The proportions of C16:0 and C18:1 fatty acids first increased and then decreased with the augmentation of NaAc concentration, whereas the C18:2 and C18:3 ratios first reduced and then rose. The optimal levels of both C16:0 and C18:1 were achieved in the treatment of 6 g/L NaAc, which were 27.41 and 21.65%, and increased by 19.64% and 1.91 times compared with the control (0 g/L), respectively. Interestingly, the minimum percentages of C18:2 and C18:3 were also observed in the 6 g/L NaAc treatment. They were 18.41 and 23.13%, and reduced by 18.50 and 35.28% in comparison with the control. This result reveals that the addition of appropriate NaAc in culture substrate could enhance the biosynthesis of C16:0 and C18:1, while the fatty acids of C18:2 and C18:3 were noticeably impeded. Zheng et al. (2017) found replenishing NaAc could obviously reduce C18:3 level in *Chlorella sorokiniana*, but C18:2 and C16:0 were significantly enhanced. And there was no dramatic change in C18:1 fatty acid. This might be contributed to the fact that the responses of algae to NaAc are species-dependent (Perez-Garcia et al., 2011).

**TABLE 2:** The fatty acid compositions of *C. reinhardtii* under different NaAc concentrations.

Fatty acid (%)	NaAc concentration (g/L)							
	
	0 (Control)	2	4	6	8	10
C14:0	1.90 ± 0.31a	1.34 ± 0.01b	0.72 ± 0.01d	0.48 ± 0.03e	0.73 ± 0.04d	0.94 ± 0.07c
C14:1	2.18 ± 0.01a	1.75 ± 0.02b	1.00 ± 0.02d	0.64 ± 0.03e	0.96 ± 0.05d	1.18 ± 0.07c
C16:0	22.91 ± 0.05e	23.76 ± 0.07d	27.40 ± 0.01a	27.41 ± 0.50a	25.19 ± 0.17b	24.13 ± 0.02c
C16:1	3.69 ± 0.01b	3.16 ± 0.03c	3.86 ± 0.07ab	4.22 ± 0.76a	4.28 ± 0.01a	4.11 ± 0.04a
C18:0	3.53 ± 0.02d	3.78 ± 0.04b	3.61 ± 0.06cd	4.07 ± 0.25a	3.69 ± 0.03bc	3.63 ± 0.05cd
C18:1	7.45 ± 0.07f	7.83 ± 0.07e	14.98 ± 0.26d	21.65 ± 0.39a	18.03 ± 0.27b	16.32 ± 0.12c
C18:2	22.59 ± 0.21b	23.30 ± 0.59a	22.61 ± 0.18b	18.41 ± 0.14e	19.42 ± 0.27d	20.18 ± 0.22c
C18:3	35.74 ± 0.38a	35.07 ± 0.53a	25.81 ± 0.61d	23.13 ± 0.26e	27.68 ± 0.58c	29.52 ± 0.40b
ΣSFA	28.35 ± 0.23d	28.89 ± 0.13c	31.74 ± 0.08a	31.96 ± 0.72a	29.61 ± 0.10b	28.69 ± 0.03cd
ΣMUFA	13.32 ± 0.07e	12.74 ± 0.08e	19.84 ± 0.34d	26.51 ± 1.12a	23.28 ± 0.21b	21.61 ± 0.15c
ΣPUFA	58.33 ± 0.17a	58.37 ± 0.05a	48.42 ± 0.43c	41.53 ± 0.40e	47.11 ± 0.31d	49.70 ± 0.18b

ΣSFA, ΣMUFA, and ΣPUFA indicate the total of saturated, monounsaturated and polyunsaturated fatty acids, respectively. The small letters in each line represent there are significant differences among varied treatments at p < 0.05. The treatment of 0 g/L NaAc was considered as the control. Data were expressed as means ± standard deviation (n = 3). The same as below.

Similarly, the maximal saturated fatty acid (SFA) and monounsaturated fatty acid (MUFA) ratios were obtained at 6 g/L NaAc treatment, where the polyunsaturated fatty acid (PUFA) proportion was lower than other treatments. The optimal levels of SFA and MUFA separately reached 31.96 and 26.51%, which promoted by 12.73 and 99.02% by contrast with the control. The minimal PUFA ratio was 41.53% and reduced by 11.84–28.85% when compared with other NaAc treatments. This result indicates that supplementing moderate NaAc in culture medium would significantly stimulate the syntheses of SFA and MUFA in *C. reinhardtii* with inhibiting the PUFA accumulation.

The above results suggest that moderately improving the carbon level of medium with adding NaAc could greatly alter the fatty acid profile of *C. reinhardtii*. The marked enhancements of SFA and MUFA could promote oxidative stability of microalgal lipids, increasing biodiesel property of *C. reinhardtii* (Knothe, 2008).

### Effects of altered carbon and nitrogen levels on the growth, lipid accumulation and fatty acid compositions of *Chlamydomonas reinhardtii*

It was found that increasing the carbon level of culture medium by supplementing moderate NaAc could dramatically enhance biomass and lipid productivity of *C. reinhardtii*, but cannot effectively trigger the total lipid synthesis in algal cells. Nitrogen concentration in culture substrate is closely related to lipid content of microalgae (Sajjadi et al., 2018). And, generally, the lipid synthesis ability of algal cells is inversely correlated with nitrogen contents. To explore a useful method for further improving the total lipid level of *C. reinhardtii* and synchronously keeping higher biomass, the influences of nitrogen limitation combined with various carbon levels on the growth and lipid production of *C. reinhardtii* were performed in this research.

#### Growth of *Chlamydomonas reinhardtii* under different carbon and nitrogen levels

**FIGURE 3:**
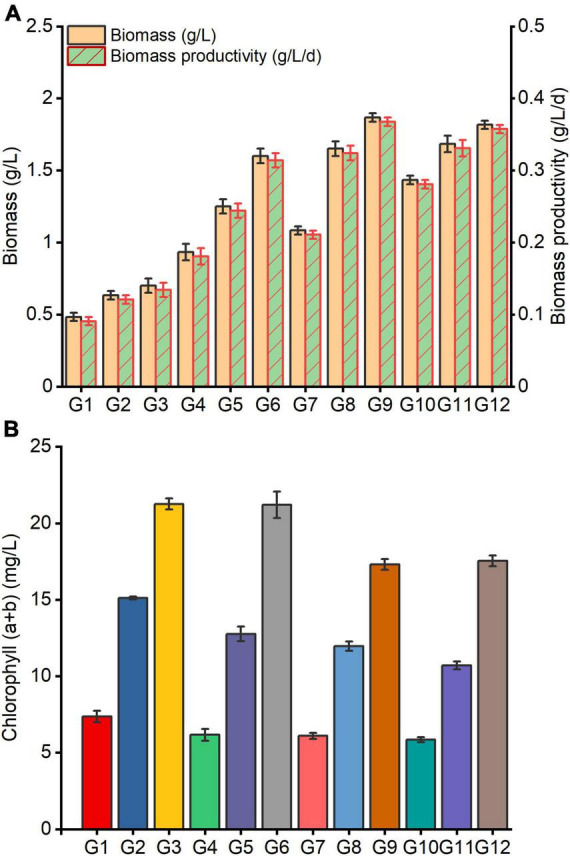
Growth of *C. reinhardtii* under different carbon and nitrogen levels. **(A)** Shows the changes of biomass and biomass productivity; **(B)** indicates the changes of chlorophyll (*a* + *b*). G1-G12 represent the combinations of different NaAc (0, 2, 4, and 6 g/L) and various NH_4_Cl (0.09, 0.19, and 0.28 g/L) in turn as shown in Table 1, and the following figures are the same.

It was observed that *C. reinhardtii* exhibited significantly different growth responses to altered carbon and nitrogen levels treatments (Figure 3). Under the same carbon level conditions, the biomass, biomass productivity and chlorophyll (*a* + *b*) content of *C. reinhardtii* were noticeably enhanced by increasing nitrogen content of medium (Figures 3A,B). Under the same nitrogen level conditions, the biomass and biomass yield rose with the augmentation of carbon content in substrate, whereas chlorophyll (*a* + *b*) displayed a declining trend (Figure 3B). The highest biomass and biomass productivity were accomplished in the treatment of G9 (4 g/L NaAc + 0.28 g/L NH_4_Cl), which were 1.87 g/L and 0.37 g/L/d, and improved by 1.67 and 1.74 times in comparison with G3 (0 g/L NaAc + 0.28 g/L NH_4_Cl) treatment. Unexpectedly, the optimal chlorophyll (*a* + *b*) content was observed at G3 treatment, which reached 21.26 mg/L. It was slightly higher than G6 (2 g/L NaAc + 0.28 g/L NH_4_Cl), and increased by 22.80 and 21.23% compared with G9 and G12 (6 g/L NaAc + 0.28 g/L NH_4_Cl) treatments, respectively.

This result reveals that the carbon and nitrogen levels of culture substrate exhibit considerable impacts on the growth of *C. reinhardtii*. Reasonably improving the carbon and nitrogen content in medium could remarkably enhance the biomass accumulation of *C. reinhardtii*. Decreasing the culture substrate nitrogen level has obviously negative impacts on the biomass production of *C. reinhardtii*. However, the noticeable improvement of the biomass was observed by supplementing carbon source in medium when nitrogen content was reduced. Compared with the changes of biomass under varied carbon levels (Figure 1A), it was found that the biomass in treated cells with 2 g/L NaAc + 0.09 g/L NH_4_Cl (G4, 0.93 g/L) was higher than that of 0 g/L NaAc treatment (0.87 g/L, containing 0.38 g/L NH_4_Cl). The biomass of treated cells with 4 g/L NaAc + 0.28 g/L NH_4_Cl (G9,1.87 g/L) was only 8.93 and 16.07% lower than 4 g/L and 6 g/L NaAc treatments, respectively, and promoted by 1.15-fold in contrast with 0 g/L NaAc treatment. This finding was also observed in other *Chlamydomonas* strains (Goodson et al., 2011; Smith and Gilmour, 2018), but many published studies were conducted under a complete lack of nitrogen source. Li et al. (2020) reported a similar conclusion in *P. purpureum* that supplying with moderate carbon source under reduced nitrogen levels can significantly improve biomass, and further enhance some active compounds yields.

#### Lipid accumulation and fatty acid compositions of *Chlamydomonas reinhardtii* under different carbon and nitrogen levels

**FIGURE 4:**
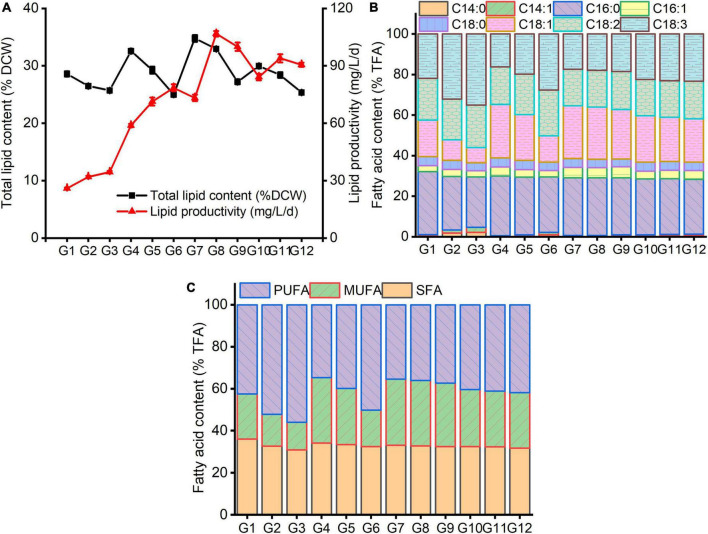
The changes of total lipids **(A)** and fatty acid profile **(B,C)** in *C. reinhardtii* under different carbon and nitrogen levels.

It can be seen from Figure 4 that, under various carbon and nitrogen levels, the marked changes of lipid content and fatty acid profile of *C. reinhardtii* were observed. Contrary to the biomass changes, under the same carbon levels, total lipid content declined with the increasing nitrogen concentration, while lipid productivity improved except for G9 and G12 treatments (Figure 4A). Under the same nitrogen content, total lipids and lipid yield increased first and then reduced with the augmentation of carbon content. A higher total lipid content was recorded at G7 (4 g/L NaAc + 0.09 g/L NH_4_Cl) treatment, which reached 34.74% and followed by G8 (4 g/L NaAc + 0.19 g/L NH_4_Cl) treatment (32.93%). It was increased by 21.61 and 47.83% compared with G1 (0 g/L NaAc + 0.09 NH_4_Cl) and 0 g/L NaAc treatment, respectively. The optimum lipid productivity (106.70 mg/L/d) was achieved under treatment of G8 and separately promoted by 2.34 and 1.71 times in contrast with G2 (0 g/L NaAc + 0.19 NH_4_Cl) and 0 g/L NaAc treatment. The lipid yield in treated cells with G7 was only 73.19 mg/L/d, which should be caused by the lower biomass (Figure 3A).

This result suggests that reducing medium nitrogen level markedly triggers lipid synthesis, and simultaneously improving carbon level could further promote lipid content of *C. reinhardtii*. Higher lipid production could be obtained by decreasing substrate nitrogen concentration combined with adding moderate carbon source. Some previous studies reported similar results that the increments of lipid production in other *Chlamydomonas* strains were observed under nitrogen-free combined with replenishing acetate (Goodson et al., 2011; Ramanan et al., 2013; Smith and Gilmour, 2018). Liu et al. (2018) probed the lipid production of *Chlorella pyrenoidosa* under varied acetate and ammonium levels, finding the addition of 2–10 g/L NaAc could significantly promote the neutral lipid content of *C. pyrenoidosa*, and lipid productivity was inclined to decrease when the concentrations of NaAc and ammonium were more than 2 g/L and 10 mg/L, respectively.

Under altered carbon and nitrogen levels of culture substrate, the changes of fatty acid compositions of *C. reinhardtii* were exhibited in Figures 4B,C. Under the same carbon level, the proportions of both C16:0 and C18:1 fatty acids reduced with the rising nitrogen content, while C18:2 and C18:3 ratios increased. The lower the carbon concentration, the more significant the difference. Under the same nitrogen level, the changes tendencies of C16:0, C18:1 and C18:2 fatty acid constituents were complicated. The C18:3 level decreased at first and then increased with the augmenting medium carbon concentration. The maximal C16:0 (31.03%) and C18:1 (26.43%) proportions were obtained at G1 and G4 (2 g/L NaAc + 0.09 g/L NH_4_Cl) treatments separately. The lowest C18:2 (17.90%) level was gained under G10 (6 g/L NaAc + 0.09 g/L NH_4_Cl). The minimum of C18:3 (16.21%) was achieved in the treatment of G4, slightly lower than G7 (17.40%) and G8 (17.90%). The minimal PUFA (34.68%) was also observed at G4 treatment, followed by G7 (35.42%) and G8 (36.06%). The optimal SFA (36.05%) and MUFA (31.51%) proportions were achieved at G1 and G7 treatments, respectively. This result reveals that reducing substrate nitrogen content coupling with adding moderate carbon source might facilitate enhancing SFA and MUFA levels with hampering PUFA production. Similar result was reported by Smith and Gilmour (2018). The decrease in PUFA ratio could improve the anti-oxidative capacity of algal lipids (Zheng et al., 2017; Ma et al., 2022).

### Optimization of culture medium carbon and nitrogen levels with response surface method

According to the above two-factor experimental results, it was found that the combination of reducing nitrogen level and increasing carbon content of culture medium could enhance the biomass and total lipid content of *C. reinhardtii* simultaneously. To obtain the optimal carbon and nitrogen contents of the culture substrate, the CCD experiment of RSM was conducted in this study. The variable levels for different runs and experimental responses were represented in Table 3.

**TABLE 3:** The variable levels for different runs and experimental responses.

Run	NaAc (g/L)	NH_4_Cl (g/L)	Biomass (g/L)	Total lipid content (%DCW)	Lipid productivity (mg/L/d)
1	6	0.28	1.85	25.32	93.66
2	2	0.28	1.65	24.68	81.44
3	4	0.32	1.95	25.52	99.50
4	6	0.09	1.10	30.80	67.80
5	4	0.19	1.70	31.98	108.73
6	6.83	0.19	1.43	25.91	74.27
7	4	0.19	1.70	32.13	109.24
8	4	0.19	1.65	32.32	106.67
9	4	0.05	0.95	34.68	65.89
10	1.17	0.19	1.27	26.49	67.09
11	4	0.19	1.65	32.16	106.14
12	4	0.19	1.70	32.05	108.97
13	2	0.09	1.05	32.15	67.53

#### Statistical analysis of central composite design

Thirteen runs were employed for analyzing the impacts of substrate carbon and nitrogen levels on biomass, total lipid content and lipid productivity. As shown in Table 3, the response of biomass concentration was 0.95–1.95 g/L, total lipid content 25.32–34.68%, and lipid productivity 65.89–109.24 mg/L/d, respectively. The significance and adequacy of the model were estimated by the analysis of variance (ANOVA) as shown in Supplementary Tables 1–3. The *p*-values of the three response models all were less than 0.0001, indicating the model terms were highly significant. The F-values for Lack of Fit of the three models were 0.3793, 0.3188, and 1.60 individually, which implied the Lacks of Fits were non-significant. The *R*^2^ values were 0.9969, 0.9994, and 0.9956, respectively. The Adeq Precision reveals the signal-to-noise ratio, which of the three responses was separately 61.3120, 135.1654, and 38.3088, suggesting the signals of the three models were adequate and could be applied to navigate the design space. According to the analyzed results, the second-order polynomial equations based on the coded factors for biomass (Eq. 1), total lipid content (Eq. 2) and lipid productivity (Eq. 3) were as follows:


(1) $$\begin{array}{l} \text{Biomass}\;\text{(g/L)}\;=\;1.68\;+\;0.0607A\;+\;0.3455B\\ \;\;\;\;\;\;\;\;\;\;\;\;\;+\;0.0375AB\;-\;0.1619A^{2}\;-\;0.1119B^{{}^{2}} \end{array}$$

(2) $$\begin{array}{l} \text{Total lipid content}\;(\%\text{DCW})\;=\;32.13\;-\;0.191A\;-\;3.24B\\ \;\;\;\;\;\;\;\;\;\;\;\;\;\;\;\;\;\;\;\;\;+\;0.4945AB\;-\;2.94A^{2}\\ \;\;\;\;\;\;\;\;\;\;\;\;\;\;\;\;\;\;\;\;\;-\;0.992B^{{}^{2}} \end{array}$$

(3) $$\begin{array}{l} \text{Lipid productivity}\;(\text{mg/L/d})\;=\;107.95\;+\;2.83A\;+\;10.91B\\ \;\;\;\;\;\;\;\;\;\;\;\;\;\;\;\;\;\;\;\;\;\;+\;2.98AB\;-\;18.40A^{2}\\ \;\;\;\;\;\;\;\;\;\;\;\;\;\;\;\;\;\;\;\;\;\;-\;12.40B^{{}^{2}} \end{array}$$

where A and B represent the levels of NaAc and NH_4_Cl, respectively.

#### Interaction of carbon and nitrogen levels on the biomass and lipid production

**FIGURE 5:**
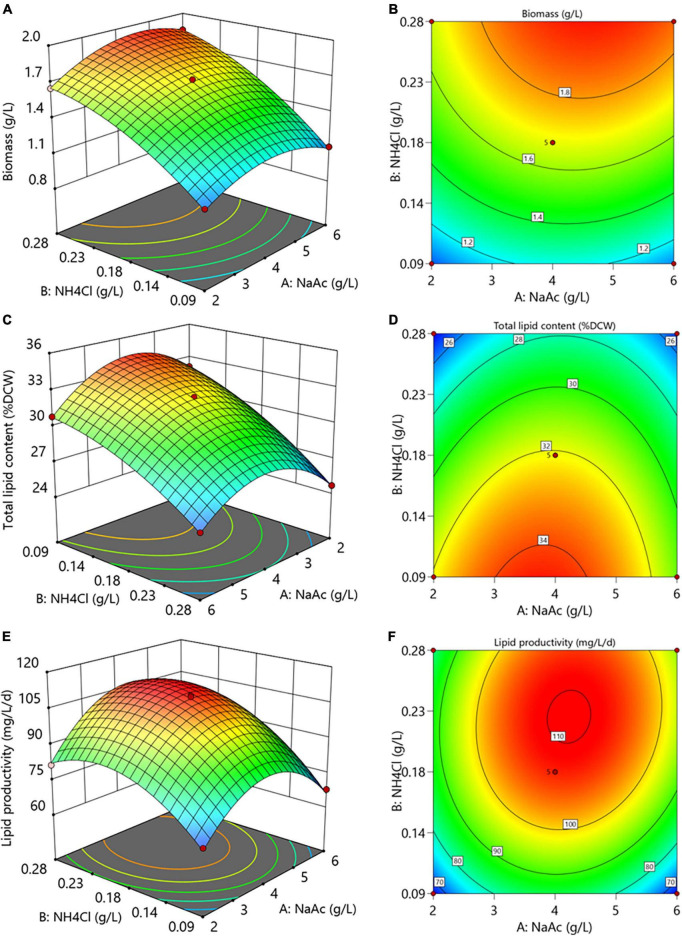
The 3D surface and contour plots of the interactions of NaAc and NH_4_Cl on the biomass **(A,B)**, total lipid content **(C,D)** and lipid productivity **(E,F)** of *C. reinhardtii*.

The interactive effects between culture medium carbon and nitrogen levels on the biomass and lipid production of *C. reinhardtii* were shown in Figure 5. The altered concentrations of NaAc and NH_4_Cl exhibited significant interactions on biomass, total lipid and lipid productivity (*p* < 0.05, Supplementary Tables 1–3). It can be seen from Figures 5A,B that appropriate NaAc and NH_4_Cl concentrations obviously enhanced the biomass, indicating that there were positive impacts of both carbon and nitrogen levels on the biomass.

However, the nitrogen content of substrate displayed a markedly opposite influence on total lipid content, which dramatically decreased with the augmentation of NH_4_Cl concentration (Figures 5C,D). Under the same nitrogen level, total lipids rose at first and then descended with the increasing NaAc concentrations. It suggests that reasonably reducing substrate nitrogen level coupled with promoting carbon concentration facilitated the accumulation of total lipid in *C. reinhardtii*. Similarly, the lipid productivity first raised and then declined with the increase of carbon and nitrogen levels (Figures 5E,F). This trend reveals that desired lipid productivity can be achieved under the conditions of moderate carbon and nitrogen concentrations. Liu et al. (2018) attained a similar result in *C. pyrenoidosa*.

#### Validation of the predicted results of central composite design

The optimal carbon and nitrogen levels predicted by the CCD models were 4.12 g/L NaAc and 0.20 g/L NH_4_Cl, in which the maximal biomass, total lipid content and lipid productivity individually reached 1.72 g/L, 31.73%, and 109.15 mg/L/d.

Based on the predicted optimum condition, the validation experiment was conducted. The results showed the validated biomass, total lipid level and lipid productivity were 1.68 g/L, 32.14%, and 108.21 mg/L/d, respectively (Table 4). Compared with the normal culture condition (TAP medium), the biomass and biomass productivity were promoted by 93.10 and 94.12%, and total lipid content and lipid productivity enhanced by 36.77% and 1.75-fold, respectively. Regarding fatty acid composition, the ratios of C16:0 and C18:1 were also remarkably improved, and C18:2 and C18:3 decreased under optimal carbon and nitrogen levels. The increments in SFA and MUFA were 17.71% and 1.28 times higher than those of TAP medium, and PUFA was reduced by 60.69%. Besides, higher intracellular soluble sugar production was observed under optimized condition, the content and productivity of which were improved by 87.87% and 2.70 times in comparison with TAP medium. In addition, the enhancement of soluble protein yield was achieved and 28.91% higher than that of the normal condition. Meanwhile, the obvious decrements in chlorophyll (*a* + *b*) and soluble protein contents were observed under optimization conditions, which was in consistent with the results reported by many similar studies (Msanne et al., 2012; Anand and Arumugam, 2015).

**TABLE 4:** The biochemical compositions and fatty acid profiles of *C. reinhardtii* under normal and optimized conditions.

Biochemical composition	Normal condition	Optimized condition	Fatty acid (%)	Normal condition	Optimized condition
Biomass (g/L)	0.87 ± 0.06b	1.68 ± 0.03a	C14:0	1.90 ± 0.31a	0.34 ± 0.04b
Biomass productivity (mg/L)	0.17 ± 0.01b	0.33 ± 0.01a	C14:1	2.18 ± 0.01a	0.42 ± 0.02b
Specific growth rate (/d)	0.67 ± 0.01b	0.81 ± 0.00b	C16:0	22.91 ± 0.05b	28.75 ± 0.22a
Chlorophyll (*a* + *b*) (mg/L)	22.34 ± 0.61a	9.94 ± 0.31b	C16:1	3.69 ± 0.01b	4.86 ± 0.21a
Total lipid content (%DCW)	23.50 ± 0.75b	32.14 ± 0.17a	C18:0	3.53 ± 0.02b	4.28 ± 0.11a
Lipid productivity (mg/L/d)	39.33 ± 1.26b	108.21 ± 1.36a	C18:1	7.45 ± 0.07b	25.05 ± 0.48a
Soluble sugar content (mg/g)	54.31 ± 1.49b	102.03 ± 3.31a	C18:2	22.59 ± 0.21a	18.04 ± 0.19b
Soluble sugar productivity (mg/L/d)	9.09 ± 0.25b	33.67 ± 1.09a	C18:3	35.74 ± 0.38a	18.26 ± 0.27b
Soluble protein content (mg/g)	130.20 ± 2.18a	85.13 ± 2.57b	ΣSFA	28.35 ± 0.23b	33.37 ± 0.09a
Soluble protein productivity (mg/L/d)	21.79 ± 0.36b	28.09 ± 0.85a	ΣMUFA	13.32 ± 0.07b	30.33 ± 0.47a
			ΣPUFA	58.33 ± 0.17a	36.30 ± 0.46b

The different lowercase letters after the values of means ± standard deviation (n = 3) in each line indicate the significant differences of various parameters under normal and optimized conditions at p < 0.05.

The above-mentioned results indicate that the flux of carbon sources might primarily turn to lipids and carbohydrate generation in the late growth stage of *C. reinhardtii* under the combination of decreasing nitrogen and increasing carbon levels (Sajjadi et al., 2018; Sulochana and Arumugam, 2020). Both sugars and proteins are high value-added active components in microalgae industrial production, which have been comprehensively utilized in food, nutraceuticals, animal feeds, pharmaceuticals, and so on (Levasseur et al., 2020; de Carvalho Silvello et al., 2022). It is well-known that the co-production of multiple active compounds is an effective strategy for decreasing the microalgae production cost and increasing the economic viability of microalgae-derived products (Rajesh Banu et al., 2020; Sarkar et al., 2020). The above results also demonstrate that the biofuel property of *C. reinhardtii* can be markedly enhanced by raising carbon level and reducing N concentration in culture medium.

## Conclusion

Microalgal lipids are greatly important lipid bio-resource and exhibit increasing prospects in many areas due to their safety, renewability, and sustainability. This study found that the obvious improvements of biomass and total lipids of *C. reinhardtii* could be simultaneously achieved by decreasing culture substrate N level combined with promoting C concentration. Under the conditions of increasing C levels *via* exogenously supplementing NaAc in culture medium alone, the maximal biomass and lipid productivity could be improved by around 1.50-fold in comparison with 0 g/L NaAc, but the total lipid content cannot be enhanced. Under independently altered N concentrations, the maximal total lipid content could be increased by 21.57% in contrast with TAP culture, but the biomass and lipid productivity separately decreased by 44.45 and 34.15%. Interestingly, the results of CCD experiment revealed that reducing ∼50% N nutrient of regular TAP substrate along with adding 4.12 g/L NaAc was the optimal N and C levels for *C. reinhardtii* to generate lipids, where the biomass, total lipids and lipid yield could be promoted by 93.10%, 36.77%, and 1.75 times compared with the cultures of TAP medium, respectively. Hence, reasonably declining culture medium N concentration and rising C supply would be an efficient method to stimulate microalgal lipid accumulation together with high biomass. The present study will be extremely important in the large-scale culture of microalgae for lipid production.

## Data availability statement

The original contributions presented in this study are included in the article/Supplementary material, further inquiries can be directed to the corresponding author/s.

## Author contributions

SZh designed the study and contributed to the formal analysis, data curation, and writing—original draft. SZo, HW, TF, and SS performed all experiments and contributed to the investigation, formal analysis, validation, and data curation. HC and QW contributed to the supervision, methodology, and writing—review and editing. All authors read and approved the final version of the manuscript.
