# Supplementary material for: Reducing culture medium nitrogen supply coupled with replenishing carbon nutrient simultaneously enhances the biomass and lipid production of Chlamydomonas reinhardtii
Source: Front Microbiol. 2022 Sep 26;13:1019806. doi: 10.3389/fmicb.2022.1019806 (PMC9549070; doi:10.3389/fmicb.2022.1019806)
Supplement: Supplementary file 1 [file Data_Sheet_1.docx]

Supplementary data

**Table S1** ANOVA for response surface quadratic model of biomass.

| Source | Sum of Squares | df | Mean Square | F-value | *p*-value |  |
| --- | --- | --- | --- | --- | --- | --- |
| Model | 1.23 | 5 | 0.2462 | 447.16 | < 0.0001 | significant |
| A-NaAc | 0.0295 | 1 | 0.0295 | 53.57 | 0.0002 |  |
| B-NH_4_Cl | 0.9551 | 1 | 0.9551 | 1735.00 | < 0.0001 |  |
| AB | 0.0056 | 1 | 0.0056 | 10.22 | 0.0151 |  |
| A² | 0.1823 | 1 | 0.1823 | 331.13 | < 0.0001 |  |
| B² | 0.0871 | 1 | 0.0871 | 158.16 | < 0.0001 |  |
| Residual | 0.0039 | 7 | 0.0006 |  |  |  |
| Lack of Fit | 0.0009 | 3 | 0.0003 | 0.3793 | 0.7740 | not significant |
| Pure Error | 0.0030 | 4 | 0.0008 |  |  |  |
| Cor Total | 1.23 | 12 |  |  |  |  |
| Std. Dev. | 0.0235 |  | *R*^2^ | 0.9969 |  |  |
| Mean | 1.51 |  | Adjusted *R*² | 0.9946 |  |  |
| C.V. % | 1.55 |  | Predicted *R*² | 0.9913 |  |  |
| Press | 0.0108 |  | Adeq Precision | 61.3120 |  |  |

**Table S2** ANOVA for response surface quadratic model of total lipid content.

| Source | Sum of Squares | df | Mean Square | F-value | *p*-value |  |
| --- | --- | --- | --- | --- | --- | --- |
| Model | 147.97 | 5 | 29.59 | 2460.55 | < 0.0001 | significant |
| A-NaAc | 0.2918 | 1 | 0.2918 | 24.26 | 0.0017 |  |
| B-NH_4_Cl | 83.87 | 1 | 83.87 | 6973.59 | < 0.0001 |  |
| AB | 0.9783 | 1 | 0.9783 | 81.34 | < 0.0001 |  |
| A² | 60.21 | 1 | 60.21 | 5005.91 | < 0.0001 |  |
| B² | 6.85 | 1 | 6.85 | 569.14 | < 0.0001 |  |
| Residual | 0.0842 | 7 | 0.0120 |  |  |  |
| Lack of Fit | 0.0162 | 3 | 0.0054 | 0.3188 | 0.8126 | not significant |
| Pure Error | 0.0679 | 4 | 0.0170 |  |  |  |
| Cor Total | 148.05 | 12 |  |  |  |  |
| Std. Dev. | 0.1097 |  | *R*^2^ | 0.9994 |  |  |
| Mean | 29.71 |  | Adjusted *R*² | 0.9990 |  |  |
| C.V. % | 0.3692 |  | Predicted *R*² | 0.9985 |  |  |
| Press | 0.2217 |  | Adeq Precision | 135.1654 |  |  |

**Table S3** ANOVA for response surface quadratic model of lipid productivity.

| Source | Sum of Squares | df | Mean Square | F-value | *p*-value |  |
| --- | --- | --- | --- | --- | --- | --- |
| Model | 4115.52 | 5 | 823.10 | 318.10 | < 0.0001 | significant |
| A-NaAc | 64.12 | 1 | 64.12 | 24.78 | 0.0016 |  |
| B-NH_4_Cl | 952.61 | 1 | 952.61 | 368.15 | < 0.0001 |  |
| AB | 35.61 | 1 | 35.61 | 13.76 | 0.0076 |  |
| A² | 2356.10 | 1 | 2356.10 | 910.56 | < 0.0001 |  |
| B² | 1068.98 | 1 | 1068.98 | 413.13 | < 0.0001 |  |
| Residual | 18.11 | 7 | 2.59 |  |  |  |
| Lack of Fit | 9.89 | 3 | 3.30 | 1.60 | 0.3217 | not significant |
| Pure Error | 8.22 | 4 | 2.06 |  |  |  |
| Cor Total | 4133.64 | 12 |  |  |  |  |
| Std. Dev. | 1.61 |  | *R*^2^ | 0.9956 |  |  |
| Mean | 88.99 |  | Adjusted *R*² | 0.9925 |  |  |
| C.V. % | 1.81 |  | Predicted *R*² | 0.9799 |  |  |
| Press | 83.19 |  | Adeq Precision | 38.3088 |  |  |
